# Advances and challenges in animal source food inspection legislation for Brazilian small-scale and artisanal producers

**DOI:** 10.1590/0102-311XEN097723

**Published:** 2024-02-02

**Authors:** Tassia Cristina Bello de Vasconcelos

**Affiliations:** 1 Ministério da Agricultura e Pecuária, Campinas, Brasil.

**Keywords:** Foods of Animal Origin, Food Safety, Food Legislation, Food Production, Alimentos de Origem Animal, Segurança Alimentar Sanitária, Legislação sobre os Alimentos, Produção de Alimentos, Alimentos de Origen Animal, Inocuidad de los Alimentos, Legislación

## Abstract

The strict sanitary inspection legislation of animal source food has been considered a trade barrier for smallholder farmers and small-scale producers in Brazil. In this sense, law flexibilization is suggested to facilitate national trade of these products. We conducted a social and sanitary analysis, presenting the current Brazilian conjuncture and difficulties for animal source food Brazilian inspection law flexibilization. By discussing inequalities, human rights issues, animal source food legislation, and international food safety standards, we evidenced critical barriers for legislative reform in Brazil. Among these barriers, the main ones are social inequalities; high zoonotic risk of animal source food products; the Brazilian political structure and its reflection on different inspection practices among country jurisdictions; and the lack of inspection services in most Brazilian municipalities. At the same time, we present positive updates in the normative framework, and point out game-changers to modify the actual safety and trade situations of Brazilian small-scale and artisanal animal source food products, including policies to strengthen state and municipal inspection services and harmonization initiatives based on international standards and national legislation. We also suggest policies to implement inspection services in municipalities, whether by municipal action or by a consortium, as well as policies to strengthen technical assistance and rural extension for small-scale and artisanal producers. These policies aim to reduce technical and sanitary education inequalities and build a fairer animal source food system.

## Resumo

A rigorosa legislação de inspeção sanitária de alimentos de origem animal tem sido considerada uma barreira comercial para agricultores familiares e pequenos produtores no Brasil. Nesse sentido, sugestiona-se a flexibilização das leis para facilitar o comércio nacional destes produtos. Realizamos uma análise sociosanitária, apresentando a atual conjuntura brasileira e apontando as dificuldades para a flexibilização da lei de inspeção de alimentos de origem animal. Discutindo as desigualdades brasileiras, os direitos humanos, a legislação brasileira sobre alimentos de origem animal e as normas internacionais de segurança de alimentos, evidenciamos pontos críticos que constituem o desafio para a reforma da legislação, sendo eles: desigualdades sociais; alto risco zoonótico de produtos de origem animal; a estrutura política brasileira e seu reflexo nas diferentes práticas de fiscalização entre as jurisdições do país; e a falta de inspeção oficial na maioria dos municípios brasileiros. Ao mesmo tempo, apresentamos atualizações positivas no arcabouço normativo e apontamos divisores de águas para modificar a real situação de segurança e comércio dos produtos de origem animal brasileiros de pequena escala e artesanais, incluindo políticas relacionadas ao fortalecimento dos serviços de inspeção estaduais e municipais e iniciativas de harmonização com base em padrões internacionais e legislação nacional; políticas de implantação dos serviços de inspeção oficial nos municípios, seja por ação municipal ou por consórcio; e políticas de fortalecimento da assistência técnica e extensão rural para pequenos produtores e produtores artesanais, em prol da redução das desigualdades na educação técnica e sanitária e construção de um sistema alimentar de origem animal mais justo.

## Resumen

La estricta legislación de inspección sanitaria de alimentos de origen animal ha sido considerada una barrera comercial para los agricultores familiares y pequeños productores en Brasil. En ese sentido, se sugiere flexibilizar la ley para facilitar el comercio nacional de estos productos. Realizamos un análisis sociosanitario, presentando la coyuntura actual brasileña y señalando las dificultades para flexibilizar la ley de Inspección de alimentos de origen animal. Al discutir las desigualdades brasileñas, los derechos humanos, la legislación brasileña sobre alimentos de origen animal y las normas internacionales de seguridad alimentaria, destacamos puntos críticos que constituyen el desafío para la reforma de la legislación, a saber: desigualdades sociales; alto riesgo zoonótico de los productos de origen animal; la estructura política brasileña y su reflejo en las diferentes prácticas de inspección entre las jurisdicciones del país; y la falta de inspección oficial en la mayoría de los municipios brasileños. A la vez, presentamos actualizaciones positivas en el marco normativo y señalamos parteaguas para cambiar la situación real de seguridad y comercio de los productos de origen animal brasileños de pequeña escala y artesanía, incluidas políticas relacionadas con el fortalecimiento de los servicios de inspección estatales y municipales e iniciativas de armonización con base en normas internacionales y legislación nacional; políticas para la implementación de servicios oficiales de inspección en los municipios, ya sea por acción municipal o por consorcio; y políticas para fortalecer la asistencia técnica y la extensión rural a pequeños productores y productores artesanos, para reducir las desigualdades en la educación técnica y sanitaria y construir un sistema de alimentos de origen animal más justo.

## Introduction

The sanitary inspection of animal source food legislation has been considered a trade barrier for smallholder farmers and small-scale producers in Brazil ^1^. The country presents a continental territory, which hinders inspection services capillarization ^2^. Moreover, the 52.9 Gini index ranks Brazil among the 10 most unequal countries worldwide ^3^, which directly reflects the enormous difficulties for small scale producers to reach great technical and sanitary levels to adhere to the Brazilian animal origin food normative framework.

Regarding the trade barrier, Nogueira Silva et al. ^1^ have conducted an analysis, highlighting two possible improvements in the Brazilian legislation to manage this issue. These include the Brazilian System of Inspection of Products of Animal Origin (SISBI-POA - Sistema Brasileiro de Inspeção de Produtos de Origem Animal) and the ARTE Label (Selo ARTE), which are considered legal flexibility and inspection capillarization tools for the national territory. Besides, the authors suggest a production scale based change in *Law n. 1,283/1950* ^4^ to allow the trade of animal products from smallholder farmers and other small scale producers across the Brazilian territory if their product is under at least one stamp of inspection, whether by municipal, state, or public consortium.

In this sense, we constructed a multidisciplinary analysis explaining the difficulties and challenges to reform *Law n. 1,283/1950* ^4^ in the current Brazilian conjuncture. Moreover, we present a historic context and updates on the legal framework of animal source food inspection produced on small scales or by artisanal production in Brazil. Complementary, we point out subjects that we understand as game-changers to modify the actual safety and trade situations of Brazilian small scale and artisanal products of animal origin in the long term.

### Challenges to the Brazilian animal source food law in a social and sanitary analysis

In the first analysis, we highlight that the human right to adequate food fits within the medical, social, and cultural rights contained in the Universal Declaration of Human Rights ^5^. It is legally internalized in Brazil by the *Federal Constitution* of 1988 ^6^ and the *Law n. 11,346/2006* ^7^, which created the Brazilian National Food and Nutritional Security System (SISAN), paving the bases for this theme in the country. According to this law, among the rights of food and nutrition security, it’s included the food safety aspect ^7^. In this sense, Brazil has *Law n. 1,283/1950* ^4^, which establishes the obligation of prior industrial and sanitary inspection, being regulated by *Decree n. 9,013/2017* ^8^, which internalizes international standards from *Codex Alimentarius*, a set of key base documents for international food safety and quality standards. The *Codex* Commission is part of a joint program of the Food and Agriculture Organization of the United Nations (FAO)/the World Health Organization (WHO) and was established with the objective of protecting consumer health and promoting fair practices in the food trade ^9^.

In view of these first considerations, it is noted that the major challenge is to guarantee the right of food safety without amplifying inequalities between large and small producers by strict sanitary rules that favor the first group due to their superior economic power and technical investments and, at the same time, impose a barrier to the smaller producers. In this sense, Nogueira Silva et al. ^1^ questioned the fact that only animal source food presents trade barriers according to the sphere of the jurisdiction, which comprises the inspection services in Brazil. In fact, *Law n. 1,283/1950* ^4^ allows the commercialization only inside the municipality that approves the inspection (not between municipalities) or only inside the state or Federal District if the product has state or Federal District seal of approval, being necessary a federal stamp for free national trade permission. This tend to occur for animal source food, with some exceptions related to equivalence and artisanal seals of approval ^4^ ^,^ ^8^ ^,^ ^10^ ^,^ ^11^ ^,^ ^12^ ^,^ ^13^ that will be discussed in this paper. In practical terms, the large-scale industry obtain federal authorization more easily, as the bureaucratic and sanitary exigences usually are perceived by producers as stricter and more expensive in the Federal Jurisdiction than in municipal or state ones.

The Brazilian legislation aims to safeguard the population right to food safety without creating trade barriers beyond those needed among jurisdictions. This principle is similar to those for different countries presented in the Agreement on the Application of Sanitary and Phytosanitary Measures (SPS Agreement) from the World Trade Organization (WTO), in which countries have the right to establish their sanitary laws, decrees, and regulations, provided that they are based on scientific principles and do not constitute arbitrary or unjustified barriers to trade, being encouraged the use of international standards, including the *Codex Alimentarius* texts ^14^. However, in Brazil, is it still necessary to keep the sanitary trade barriers for animal source food according to the jurisdiction level of inspection? Why, despite municipal and state inspection services for animal source foods, these products are still prohibited to be sold throughout the national territory, differently from non-animal source foods products according to *Decree-Law n. 986/1969* ^15^?

These questions must be answered carefully part by part. In the first scenario, it is important to note that stricter legislation for animal products is centered on high zoonotic risk, besides the possibility of veterinary drug residues in animal source food, points that are corroborated by the World Organization for Animal Health (WOAH) for products of animal origin ^16^. In fact, 60% of pathogens that cause human diseases originate from animals ^17^ and, for example, bacteria such as *Salmonella*, *Campylobacter*, and *Escherichia coli* are common foodborne pathogens that affect millions of people each year, with severe and fatal outcomes ^16^. This situation is more complex to be handled in a continental and unequal country such as Brazil ^3^. In a second point, it is important to highlight that Brazil is a Federative Republic, where a Federative Pact in force gives legislative autonomy to municipalities and states ^5^. Although federal legislation must prevail over states and municipalities ^5^, in practice, some differences may be found between inspection of animal source food in each autonomous jurisdiction, also considering the immense diversity of political parties existing in the country (nowadays, 31 parties) ^18^, which can command each location. Moreover, unfortunately, variations in microbiological quality have already been demonstrated among products under different jurisdiction inspection in the scientific literature, showing better results, for example, in colony cheese under federal inspection when compared with others from state or municipal ones, in some assessments ^19^. For these reasons, it is still necessary to be very careful when evaluating changes in the law, to guarantee food safety without neglecting the Brazilian continental territory and its unequal structure.

## Brazilian initiatives to animal source food inspection harmonization, a normative update

### The SISBI-POA initiative

To harmonize the distortions presented in this paper, *Decree n. 5,741/2006* ^20^ regulated the Unified System of Attention to Agricultural and Livestock Health (SUASA - Sistema Unificado de Atenção à Sanidade Agropecuária) and determined that the entities of the country’s jurisdictions must provide measures that favor animal source food inspection in a uniform, harmonious, and equivalent way in all states and municipalities ^20^. In this sense, SISBI-POA was created by the Brazilian Ministry of Agriculture to provide a unified and equivalent inspection system for states, federal district, and municipalities (or municipal public consortiums) ^10^. This system works by equivalence recognition by federal audits and SISBI-POA seal of approval to allow national trade ^21^. The System considers yet two *Normative Instructions* (NI) for small agro-industrial establishments: *NI n. 05/2017* ^22^, which establishes the requirements for assessing equivalence in terms of physical structure, facilities, and equipment; and *NI n. 16/2015* ^23^, which establishes, throughout the national territory, specific standards for inspection of animal source food to small agroindustries ^23^. Therefore, we understand that, in view of current inequalities described in this analysis, policies for strengthening the SISBI-POA are a game-changer to improve equity in the Brazilian inspection animal source food system due to allowing national trade of animal source food products, including those from small-scale producers. This seal of approval considers equivalence and both quality and safety of animal source food products following the Brazilian Ministry of Agriculture standards.

### Initiatives for artisanal seals of approval

Other important initiatives include the ARTE Label and the Artisanal Cheese Label (Selo Queijo Artesanal), created, respectively, by *Law n. 13,680/2018* ^11^ and *Law n. 13,860/2019* ^12^. These labels indicate not only sanitary condition adherence, but also products that have unique and differentiated organoleptic properties, including those produced in an artisanal way related to region, tradition, or culture ^13^. Initially, the national trade of these artisanal products was allowed by Public Health state or federal district control, but recently, *Ordinance n. 531/2022* ^24^ amplified this control for all jurisdictions (by Municipal, State, Federal District, or Federal controls). It is important to highlight that *Law n. 13,680/2018* is already an alteration inserted in *Law n. 1,283/1950*, changing the jurisdiction control and allowing national trade for artisanal products ^13^. However, considering the inequality conjuncture already presented in this analysis, *Ordinance n. 531* ^24^ maintains the double check by federal jurisdiction, by an audit system of the Brazilian Ministry of Agriculture. Therefore, we believe that policies for strengthening Artisanal Labels are a game-changer to improve equity in the Brazilian inspection animal source food system, without neglecting to consider the current inequalities of Brazilian conjuncture that still require greater care, for example, with double-check systems, to guarantee food safety for the population.

### Commission for Traditional Foods of the Peoples of Amazonas and Catrapovos Brasil initiatives

Traditional Indigenous peoples are classified as family farming ^2^ ^,^ ^25^. In this context, in 2015, *Decree n. 8,471* modified the SUASA Regulation (*Decree n. 5,741/2006*), allowing a different perspective on small-scale producers and family farming. This modification allowed to classify the agro-industrial establishment of animal source food products as artisanal agro-industry under the condition that these products derive from customs, habits, and/or traditional knowledge, aiming to value food diversity and multiculturalism of peoples, traditional communities, and family farmers ^26^.

In this sense, the Commission for Traditional Foods of the Peoples of Amazonas (CATRAPOA - Comissão de Alimentos Tradicionais dos Povos no Amazonas) initiative has been working since 2016 in the Amazonas State, articulating Federal, state, and municipal governments, Indigenous movements and leaders, traditional communities, and civil society organizations ^27^. CATRAPOA aims to be a permanent forum for discussions, articulations, and actions on traditional foods ^28^. Based on the CATRAPOA experience, in 2021, by *Ordinance n. 16/2021/6CCR/MPF*, februray 11, 2021, the Catrapovos Brasil Permanent Dialogue Table (Mesa de Diálogo Permanente Catrapovos Brasil) was established with the objective of promoting dialogue and integration between government and civil society bodies related to the topics of traditional peoples and communities at the national level, public procurement, and food and nutrition sovereignty and security. It also aimed to be a space to discuss possible adjustments to actions and regulations linked to the topic and encourage the implementation of regionalized school meals and other public purchasing mechanisms appropriate to the culture of traditional peoples and communities across different regions and states of Brazil via local arrangements ^29^.

In this context of food discussion, for a proper understanding of the condition of the Indigenous peoples food, it is important to be aware that both *Decree n. 8,471/2015* ^26^ and *NI n. 16/2015* ^23^ (related to inspection in small-scale agro-industry) exempt registration and inspection of agricultural products for family consumption, which, as previously stated, wich includes Indigenous peoples’ case ^2^ ^,^ ^30^. It occurs because the entire production and food preparation process generally occurs within the family community, as does school meals for students ^31^. However, it is important to highlight that this condition does not neglect the health needs inherent to the issue but still considers that Indigenous and traditional peoples have their own ancient mechanisms for preserving and handling food, and are capable of guaranteeing a minimum quality for consumption in the local environment of Indigenous Lands ^30^.

This thesis is supported by *Technical Note n. 01/2017/ADAF/SFA-AM/MPF-AM* ^30^ by several anthropological studies and national and international documents, such as the *Brazilian Federal Constitution* of 1988 and the United Nations International Convention on Biological Diversity/1992, which defend the family farming and the Indigenous food, as well as their own processes and local practices ^2^ ^,^ ^30^. Subsequently, in 2020, *Technical Note n. 3/2020/6CCR/MPF* ^32^ was issued, which extended the understanding to all traditional peoples and communities in Brazil. It stated that state health standards must serve as a reference for the preparation, handling, and storage of food but traditional peculiarities must be respected.

*Technical Note n. 01/2017/ADAF/SFA-AM/MPF-AM* ^30^ also presents specific considerations about Indigenous school feeding. For example, the issue related to items delivery that did not meet the cultural demands of local people have been reported to affect Indigenous peoples’ health and their environment due to the supply of processed products accompanied by plastic packaging. In this context, the Technical Note was issued to improve Indigenous peoples’ access to different food, respecting their own traditional production processes, including hiring school meals, under the terms of the Brazilian National School Feeding Program (PNAE). This program was established by *Law n. 11,947/2009* ^33^, with a minimum mandatory acquisition of 30% of meals directly from family farming and rural family producers and their organizations, prioritizing agrarian reform settlements and traditional and Indigenous communities.

For this issue of school feeding, in 2020, a practical guide on Indigenous school meals and traditional communities was launched. An Indigenous and traditional school feeding strategy in Amazonas was developed by the CATRAPOA initiative, and the guide is supported by the Green Markets and Sustainable Consumption Project, an initiative of the German Federal Government, in partnership with the Brazilian Ministry of Agriculture and with support from the private consortium ECO Consult Sepp & Busacker Partnerschaft, a non governmental scientific organization ^27^.

### Brazilian barriers to advancing in the harmonization process and the need for specific policies for municipalities and states

In general, the animal source food inspection system for small-scale and artisanal production in Brazil presents limitations, including jurisdiction differences found across regions on animal source food inspection, as previously discussed, and the lack of municipal inspection services, which is established in few municipalities ^34^. Therefore, before integrally changing the issue of trade and jurisdiction control responsibility in *Law n. 1,283/1950* ^4^, other important policies must be implemented. These could include policies to strengthen state and municipal inspection services that already exist, also considering actions related to harmonization of inspection methodology based on international or national standards, such as *Codex Alimentarius*, as well as policies aimed at implementing municipal inspection services in municipalities, whether by municipal action or by consortium.

### Brazilian actions based on the farm to fork WOAH strategy, a normative update

It is important to highlight WOAH considerations about the need for actions in all stages of the food chain, from production at farm to human consumption. In fact, control of the primary production at the farm is important to reduce the burden of animal disease and risk of human illness via foodborne contamination ^16^. In this sense, the Brazilian normative framework established rules of both good agricultural practices in the farm and good manufacturing practices in the production. For example, for good agricultural practices, *NI n. 73/2019* presents Technical Regulation of Good Agricultural Practices aimed at rural producers who supply milk for the manufacture of artisanal dairy products, with *Codex Alimentarius* standards incorporation ^35^, such as the Code of Hygienic Practice for Milk and Milk Products (CAC/RCP 57-2004) ^36^. Another example is *NI n. 61/2020*, which presents the regulation for the framework of meat and artisanal products, with both good agricultural practices and good manufacturing practices citations ^37^. Besides, the *Decree n. 9,013/2017*, established by the Regulation of Industrial and Sanitary Inspection of Products of Animal Origin (RIISPOA - Regulamento da Inspeção Industrial e Sanitária de Produtos de Origem Animal), the most important document for animal source food inspection in Brazil, also brings incorporations of *Codex Alimentarius* standards ^8^, such as the General Principles of Food Hygiene (CXC 1-1969) ^38^.

The Brazilian inequality scenario is a major barrier to the Farm to Fork Strategy. In this sense, Brazilian policies must offer conditions to small producers to improve their good agricultural practices and good manufacturing practices, which we understand as strongly linked to technical assistance and rural extension policies, the last game-changers in this analysis. In this context, the *Brazilian Law of Agricultural Policy n. 8,171/1991* mentions rural extension as an agricultural policy instrument ^39^, and some Brazilian laws and guidelines for artisanal products also cite these actions (*NI n. 73/2019* and *n. 61/2020*; *Law n. 13,860/2019*; and *Ordinance n. 531/2022*) ^12^ ^,^ ^24^ ^,^ ^35^ ^,^ ^37^. However, in practice, an immense need for investment in rural extension policies is still present across the Brazilian territory in favor of granting less unequal animal source food production. Therefore, policies for strengthening technical assistance and rural extension, which allow the implementation of these actions in the national territory, are game-changer for building a fairer animal source food system.

### Other complementary ways to overcome the sanitary exclusion in Brazil

As previously discussed, the Brazilian heterogenic political frame ^18^, with variations in each location government, as well as the picture of national inequality ^3^, are critical points that hinder sanitary inclusion in the country. In this context, it is important to highlight that the four game-changers presented in this discussion come up against major obstacles to reach their aims, requiring complementary public policies in a multidisciplinary and wide field, which constitutes by itself an immense challenge.

First, policy programming is hindered due to lack of data in the work field, which is the case for most of the 5,570 municipalities in Brazil ^40^. For example, there is no data on the exact number of municipalities with animal source food inspection system. Therefore, for strengthening and establishing harmonized inspection initiatives and services, such as SISBI-POA and ARTE Label, diagnostic analyses must be conducted for each local and/or regional reality. It is necessary to reduce all types of inequalities in Brazil to promote food security and sustainable agriculture ^41^. In this sense, due to the many inequalities that exist (ethnicity, wealth, and access to education and healthcare, for example) and Brazil’s continental size and cultural heterogeneity, data must be continuously collected at the local and/or regional level to better know and handle each reality. Therefore, it is urgent to strengthen data collection bodies such as the Brazilian Institute of Geography and Statistics (IBGE), as well as research, teaching, and extension centers that can contribute strongly to data collection ^2^ in cooperation agreements with local, state, and federal governments.

Secondly, it is important to consider the cultural and traditional people issue, as previously discussed, which requires strengthening institutions such as the Brazilian National Institute of Historic and Artistic Heritage (Iphan - Instituto do Patrimônio Histórico e Artístico Nacional) and the Brazilian National Indigenous People Foundation (FUNAI - Fundação Nacional dos Povos Indígenas), especially in the relationship between anthropology and the State as a technical work tool ^2^.

Lastly, it is important to note the history of Brazilian public policies for food and nutrition security, as well as for the reduction of social inequalities. Historically, actions that aimed to promote food accessibility, healthy diet, dialogue between family farming and food and nutrition security, agroecology, and the valorization of local territories and food have been fragile and unstable, as well as sensitive to political and economic changes. For example, from 2000 to 2015, a conflicting coexistence between productivism and food and nutrition security was noted; and from 2014 to 2022, a dismantling sectoral referential ^42^.

Regarding contradictions noted in 2000 to 2015, a strong investment in the primarization of economies was noticed, by commodities export, versus policies based on the expansion of freedom and rights of the poorest and on inclusive institutions. Despite these facts, it is important to highlight that policies were established or strengthened for food production and enhancement of food accessibility and security, including technical assistance and rural extension, land credit, as well as the creation of the Food Acquisition Program, the National Policy and Plans for Agroecology and Organic Production, and poverty reduction initiatives in general ^42^.

The Brazilian political scenario has recently changed with the return of the political party that was mostly in charge from 2000 to 2015. Therefore, based on a historical analysis, the reconstruction and strengthening of food and equality policies is expected. We highlight that this is also a critical moment to demand these policies from the stakeholders in this field who are currently on the government chairs.

## Conclusions

In conclusion, before promoting new changes to the issue of trade and jurisdiction control responsibility in the Brazilian animal source foods *Law n. 1.283/1950*, a set of issues must be addressed to promote a fairer animal source food inspection system, including: (1) strengthening of inspection harmonization initiatives across Brazilian jurisdictions, such as SISBI-POA and ARTE Label, with a double-check federal audit process; (2) policies related to strengthening state and municipal inspection services that already exist, including harmonization methodology based on international standards and national legislation; (3) policies to implement municipal inspection services, whether by municipal action or by consortium, where they do not exist yet; and (4) Policies to strengthen technical assistance and rural extension throughout the national territory for small-scale and artisanal producers to reduce technical and sanitary education inequalities.
